# Comparison of Cu Strengthened by Ionic Bonded Particles and Cu Strengthened by Metallic Bonded Particles

**DOI:** 10.3390/ma18112648

**Published:** 2025-06-05

**Authors:** Ke Han, Vince Toplosky, Rongmei Niu, Yan Xin

**Affiliations:** National High Magnetic Field Laboratory, Florida State University, Tallahassee, FL 32306, USAniu@magnet.fsu.edu (R.N.); xin@magnet.fsu.edu (Y.X.)

**Keywords:** high strength conductor, cryogenic properties, mechanical strength, cyclic loading, fatigue, dispersion strengthening

## Abstract

Cu matrix composites, because of their high mechanical strength, are often used as conductors in high-performance electrical applications. These composites are manufactured through thermomechanical processing, which introduces a high density of particles that act as obstacles to dislocation motion. Increasing the density of these particles enhances the mechanical strength of the conductors, which we tested under static loading. Under cyclic loading, especially pulsed electrical mechanical loading, conductors may soften, harden, or even fail. Failure is likely to occur whenever the applied stress exceeds the flow stress of the conductors. Understanding and predicting the performance of conductors under cyclic loading can help researchers estimate the lifespan of any apparatus made from these conductors. The performance of conductors depends on whether the strengthening particles are characterized by ionic interatomic bonding or metallic bonding. During fabrication, we observed both the accumulation of dislocations and the dissolution of particles (which added more solute atoms to the matrix). Because both dislocations and solute atoms tend to migrate at room temperature or higher, the complexity of microstructure changes increases in composites under cyclic loading. To minimize such complexity, we designed our test to determine fatigue properties at 77 K. We subjected the conductors to cyclic fatigue tests using a load-controlled mode (the mode most commonly used in applications). This work sheds light on the correlation between tensile properties and fatigue properties in our composite conductors. We found that the correlation varied, depending on whether the conductors had been strengthened by ionic bond or metallic bond particles.

## 1. Introduction

Copper is an important conductor due to its excellent electrical conductivity and low cost. However, its modest strength limits its applications. Although alloying copper through a solid solution can enhance its strength, this process results in a reduction in electrical conductivity. Therefore, researchers have developed various composites that are strengthened by particles, which impede dislocation motion in copper, thereby increasing its strength [1,2,3,4,5,6,7,8,9,10,11].

When a dislocation is impeded by a particle, the dislocation can either shear the particle or bow around it to continue its movement. In the first case, the particles are usually soft, so they are referred to as “soft particles” in this paper. The atomic bonding in these soft particles is metallic in most cases, although it can be ionic. In this paper, we focused on soft particles with metallic bonding. In the second case, since the particles are usually hard, they are referred to as “hard particles”. The atomic bonding in these particles can be either metallic or ionic. In this paper, we focused on hard particles with ionic interatomic bonding.

Ceramic particles, typically characterized by ionic interatomic bonding, are generally hard and resistant to shearing by dislocations in copper (Cu). Alumina is one such ceramic used to strengthen Cu. A Cu–alumina composite can be produced from Cu-Al alloy powders that undergo internal oxidation to form alumina particles [12]. A commercially available Cu–alumina composite is known as GLIDCOP^®^, a trademarked line of alumina-strengthened copper conductors. These conductors are favored for use in pulsed magnets and fusion applications [13,14,15,16,17,18,19,20,21,22,23,24,25,26].

Metallic particles, especially those with a crystallographic structure similar to copper (Cu), can be sheared by dislocations in Cu. Chromium (Cr) is one such metallic strengthening particle. A commercially available Cr-particle-strengthened Cu alloy is C18150, which is typically produced by casting.

Most C18150 and GLIDCOP^®^ billets undergo further processing through extrusion and deformation techniques. This approach helps reduce the size of voids and enhances both ductility and fracture toughness [27].

For large-sized conductors, both alumina- and chromium-strengthened copper (Cu) conductors are stronger than pure Cu [28,29,30,31,32,33] and exhibit high creep resistance [34].

Ceramic-particle-strengthened copper (Cu) performs well at temperatures above ambient. For example, GLIDCOP^®^ retains its high strength at temperatures up to 500 °C [35]. Long-term exposure to high temperatures has a minimal impact on its microstructure [36,37]. In contrast, metallic-particle-strengthened Cu, such as chromium (Cr)-particle-strengthened Cu, can only retain its strength at temperatures below 300 °C [32,33].

Heat treating a deformed composite at high temperatures decreases mechanical strength and increases ductility in particle-strengthened conductors, even when the particles are made of ceramics [38].

Deformation strengthens particle-strengthened copper (Cu) [39]. Because alumina is harder than chromium (Cr) and Cu, it is more susceptible to cracking in stress-concentrated regions. Therefore, cold deformation of alumina-strengthened Cu to a high degree of strain is more problematic for GLIDCOP^®^ than for Cr-particle-strengthened Cu [40,41]. Deformation results in the formation of cracks and an increase in the number of pores, followed by the decohesion of small alumina particles. The final fracture path is the result of the coalescence of previously originated cracks [17]. Such decohesion has not been reported in Cr-strengthened Cu, provided that the Cr particles are small and the Cr content is below 1 wt% [32,33].

Deforming copper (Cu) matrix composites introduces anisotropy. For example, in extruded samples of alumina-strengthened Cu, mechanical strength and fatigue resistance are higher in the extrusion direction than in the transverse direction [39].

All mechanical properties of composites are governed by the size and distribution of their particles. In both types of composites, different researchers have reported varying particle sizes, ranging from 3 nm to 1 µm [32,33,36,42]. This inconsistency in reported particle sizes highlights the need for further studies.

In addition to static mechanical loading, it is crucial to understand conductor performance under cyclic loading. Accurate knowledge of fatigue properties helps avoid catastrophic and expensive failures and optimize estimates of component life. In fatigue tests, annealed GLIDCOP^®^ samples exhibit both cyclic hardening and softening, whereas as-deformed samples typically show softening [13]. Because dislocation substructures are stabilized by alumina particles after deformation, softening occurs much more slowly in GLIDCOP^®^ than in pure copper (Cu). Therefore, at high stress ranges, the fatigue life of GLIDCOP^®^ should be longer than that of Cu. At low amplitudes, the fatigue lives of both materials are similar [16]. Similar arguments apply to Cr-particle-strengthened Cu.

Our work, which focused on particle type, particle size, and particle distribution in copper (Cu) matrix composites, revealed a much larger particle size distribution than previously reported by researchers in this area. Our work revealed certain reasons for this inconsistency and analyzed the impact of the strengthening particles on the properties of Cu matrix conductors.

**Figure 1:**
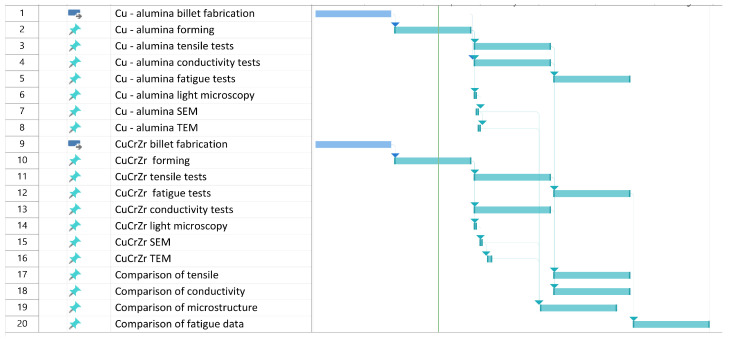
Gantt chart of current work. “CuCrZr forming” and “Cu- alumina forming” indicate that materials have been deformed by either swaging or cold drawing. “Tensile” indicates that materials have been subjected to tensile tests and analyses.

In this paper, we first establish the relationship between mechanical strength and electrical conductivity at both room temperature and 77 K to set a baseline for our samples. Following this, we report the plastic deformation behaviors of materials under cyclic loading. We then compare the microstructure of two types of conductors, examining them from the microscale to the atomic scale. In the discussion, we link the material deformation behavior to the observed microstructure (Figure 1).

## 2. Materials and Methods

The conductor rods used in this study were originally fabricated from six different billets, each weighing at least 150 kg. Four of these billets were Cu+alumina (GLIDCOP^®^ Al60), and two were CuCrZr. The GLIDCOP^®^ Al60 rods with pure Cu cladding were acquired in extruded and deformed (Ex+Def) condition. The nominal alumina content in the Al60 core of the rods was 1.1 wt.% (1.62 vol.%). CuCrZr rods were acquired in forged, extruded, and deformed (For+Ex+Def) condition, with nominal contents of Cr and Zr being 0.5 wt.% and 0.05 wt.%, respectively.

We further deformed all the conductor rods at room temperature into a rectangular shape (6.7 × 11 mm^2^ in cross-section with 1.6 mm corner radii) and divided them into six batches (Figure 1).

Conductivity measurements were performed on at least three samples from each batch, each with a nominal length of 150 mm. Conductivity was measured using a standard four-point method: current leads were clamped onto the ends of each sample, and voltage taps were clamped onto the samples at two midpoints approximately 100 mm apart. The values reported here are estimated to be accurate within ±1% of the International Annealed Copper Standard (IACS).

Mechanical tests were conducted on a 100 kN servo-hydraulic MTS test machine (MTS Systems, 14000 Technology Drive, Eden Prairie, MN, USA) equipped with a cryostat, allowing specimens to be immersed in liquid nitrogen. These tests were performed at room temperature (approximately 295 K) and at 77 K. For each batch at each temperature, we used at least three samples. In tensile tests, the samples were loaded at a displacement control rate of 0.5 mm/min. An unload/reload cycle was performed to determine the elastic modulus and analyze plastic deformation behaviors. A 25 mm gage-length extensometer (3442-25M-005-LHT, Epsilon, Technology Corp., Jackson, WY, USA) was used to record strain, and a 100 kN load cell to measure force. Because our Cu–alumina conductors had cladding at the surface, we used both full cross-section and reduced cross-section samples. Most of the data reported in this paper were from the full cross-section samples. The tests were performed following standard test procedures ASTM E8 [43] and ASTM E1450 [44].

**Figure 2:**
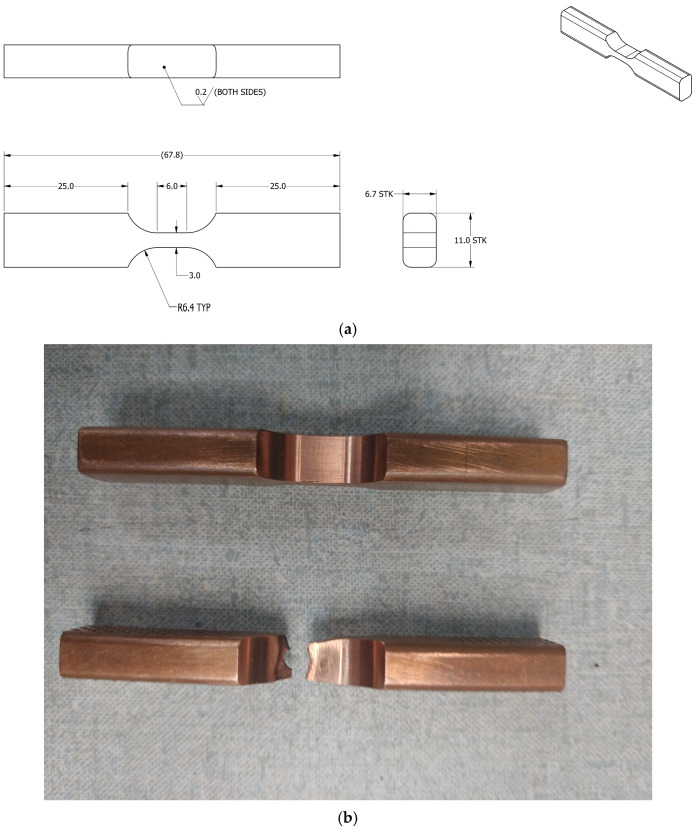
Sample geometry for cyclic tests: (**a**) Sample geometry (units in millimeters). (**b**) Image of samples before (**top**) and after (**bottom**) a test.

Fatigue tests were conducted at 77 K. To reduce stress concentration, fatigue samples were machined into a flattened hourglass shape with a nipped-in waist. Strain values were recorded using a diametric extensometer (Figure 2).

After initially examining the microstructure of polished samples using a light microscope, we re-examined these samples under a Focused Ion Beam (FIB) Scanning Electron Microscope (SEM) (Thermo Fisher Helios G4 UCym, Thermo Fisher Scientific Inc., 168 Third Avenue, Waltham, MA, USA). Samples for this SEM were sectioned from the conductors to provide either transverse or longitudinal views, or both. When acquiring images or preparing samples using an ion beam of Ga, we set the gun to high voltage (30 kV) with a current of 24 nA. For acquiring images using a backscattering electron detector, we set the gun to low voltage (2 kV) with a current of 1.6 nA.

We studied strengthening mechanisms from the longitudinal perspective using a JEOL JEM-ARM200cF Transmission Electron Microscope (TEM, JEOL USA, Inc., 11 Dearborn Road, Peabody, MA, USA) equipped with an Oxford Aztec Energy-Dispersive Spectroscopy (EDS) detector (300 Baker Avenue, Suite 150, Concord, MA, USA). Some TEM specimens were prepared by grinding them to a thickness of about 50 μm, from which a 3 mm diameter disk was punched out and subsequently ion- or jet-polished. Other samples were prepared using FIB. The major imaging technique used in this study to analyze strengthening particles was High-Angle Annular Dark Field Scanning Transmission Electron Microscopy (HAADF-STEM). The STEM resolution of our microscope was 0.78 Å. Data for Energy-Dispersive X-ray Spectroscopy (EDS) were collected in STEM mode with a probe size of 0.11 nm using a Gatan GIF (Las Positas Blvd. Pleasanton, CA, USA) and an Oxford Aztec EDS detector (Figure 1).

## 3. Results

In this study, we aimed to link microstructural characteristics to mechanical strength, electrical conductivity, and fatigue properties in two types of conductors: Cu strengthened by metallic bonded particles and Cu strengthened by ionic bonded particles. We reported both electrical conductivity and mechanical strength, as these properties have direct and indirect impacts on material performance under cyclic loading. To understand the differences in mechanical properties between the two types of conductors, we carefully compared their respective microstructures.

### 3.1. Tensile Properties and Electrical Conductivity

Room-temperature test results showed that our six batches of conductors had ultimate tensile strength (UTS) values ranging from 535 to 590 MPa and electrical conductivity (EC) values ranging from 82 to 84% IACS. Consistent with previous research, we found that conductors with higher mechanical strength exhibited lower electrical conductivity. For example, samples from the conductor with an average UTS of 590 MPa showed an EC of 82% IACS, while samples from the conductor with an average UTS of 535 MPa showed an EC of 84% IACS. Previous researchers have utilized these properties in the construction of various facilities designed to optimize conductor performance. In the 100 T pulsed magnet at the National High Magnetic Field Laboratory (NHMFL), engineers, aware that the highest mechanical stress would be generated at the magnet’s center, chose to construct the insert from conductors with the highest available mechanical strength, despite their relatively lower EC. To prevent overheating in regions with relatively lower stress, they used conductors with intermediate strength levels but high electrical conductivity [45]. Among those conductors, two batches of CuCrZr exhibited higher strength, with one batch showing the highest UTS but the lowest EC at room temperature.

**Figure 3:**
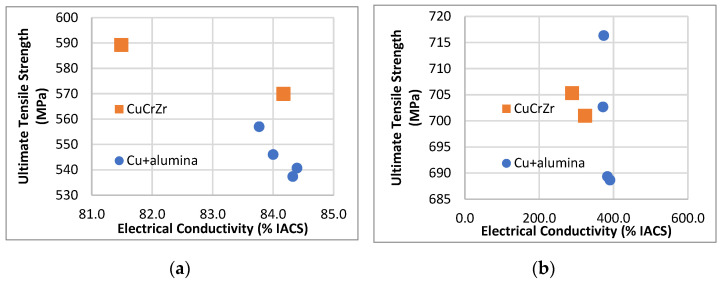
Graphs showing ultimate tensile strength versus electrical conductivity for samples representing two types of composites. Each dot in the graph represents at least six tests for electrical conductivity and mechanical strength. Round dots represent Cu–alumina, and squares represent CuCrZr. (**a**) Data obtained from tests performed at room temperature. (**b**) Data obtained from tests performed at 77 K.

At 77 K, the conductivity of Cu–alumina composites was generally higher than that of CuCrZr, although the strength-versus-conductivity trend was not entirely clear. Three of the four batches of Cu–alumina composites showed lower UTS at 77 K compared to the two batches of CuCrZr, while the UTS of the fourth batch fell between those of the two CuCrZr batches (Figure 3).

To prepare for characterizations of samples under cyclic loading, we selected samples from a batch of CuCrZr and a batch of Cu–alumina, two composites that exhibited nearly identical EC at room temperature (Table 1). At 77 K, CuCrZr exhibited lower EC than Cu–alumina, indicating that CuCrZr has a higher concentration of alloying elements dissolved in its solid solution.

At room temperature and at 77 K, the two composites showed almost the same elastic constant and less than a 10% difference in mechanical strength. CuCrZr exhibited a higher reduction-in-area at fracture compared to Cu–alumina, indicating greater ductility in CuCrZr (Table 1).

**Figure 4:**
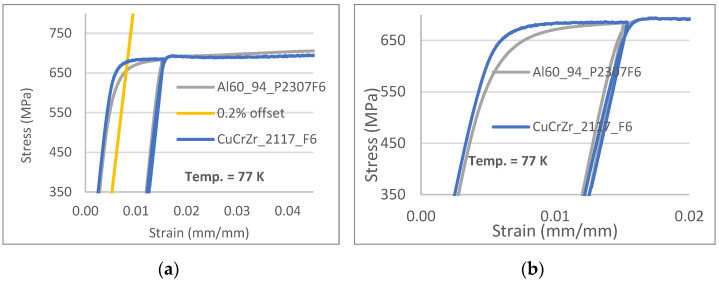
Comparison of stress–strain curves for Cu+alumina (thin grey lines) and CuCrZr (thick solid blue lines), tested at 77 K. (**a**) Stress–strain curves with tensile stress above 350 MPa. (**b**) Stress–strain curves with total strain below 2%.

Above a strain level of 0.02 (2%), samples of Cu–alumina tested at 77 K exhibited a higher strain-hardening rate, resulting in approximately 1% higher ultimate tensile strength (UTS). A close examination of the stress–strain curves for strain values below 0.02 (measured at 77 K) revealed differences in plastic deformation behavior (Figure 4). Samples of Cu–alumina began to undergo plastic deformation earlier than samples of CuCrZr, resulting in lower flow stress in Cu–alumina at strains below 0.015.

### 3.2. Properties Under Cyclic Loading

We performed our tests primarily under asymmetric tension–compression cyclic loads, designed to produce higher absolute values for tensile loading than for compression. Throughout each series of tests, the relationship between tensile and compressive loads remained consistent from beginning to end. We tested each sample until failure, with most samples surviving more than 5000 cycles. The highest levels of plastic deformation strain appeared in each sample during its first half-cycle.

**Figure 5:**
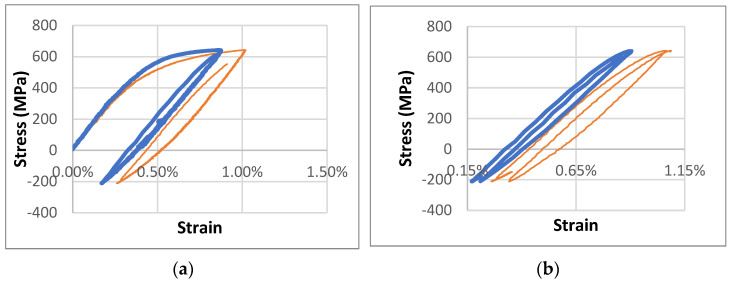
Stress–strain curves at 77 K for a Cu+alumina sample (thin orange solid line) and a CuCrZr sample (thick blue solid line) under a maximum load of 643 MPa and minimum load of 211 MPa. (**a**) Comparison of curves during the first cycle. Plastic deformation strain amplitude values are approximately 0.37% and 0.51% for CuCrZr and Al60, respectively. (**b**) Comparison of curves during the second cycle. Plastic deformation strain amplitude values are approximately 0.08% and 0.16% for CuCrZr and Al60, respectively.

Our first sequence of tests was set for a tensile load of 643 MPa and a compressive load of 211 MPa, ensuring that (1) the stress amplitude level matched the average tensile strength and (2) the maximum stress level fell between the yield strengths of CuCrZr and Cu–alumina. Using dimetric strain measurements, we studied the differences in plastic deformation behavior under cyclic loading between CuCrZr and Cu–alumina. We found that the plastic deformation strain amplitude of CuCrZr at the end of the first half-cycle of loading was about 70% of that of Cu–alumina. At the end of the second full cycle of loading, the plastic deformation strain of CuCrZr was only about 50% of that of Cu–alumina (Figure 5).

**Figure 6:**
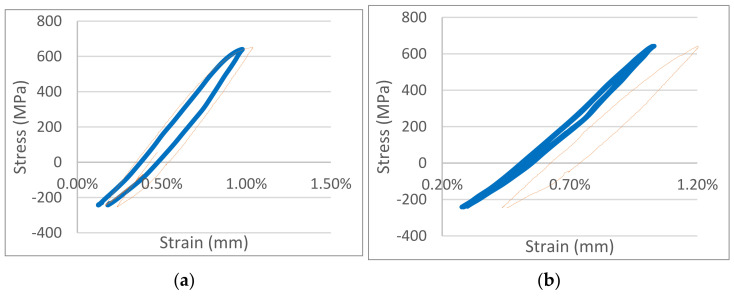
Stress–strain curves at 77 K for a Cu+alumina sample (thin orange solid line) and a CuCrZr sample (thick blue solid line) under a maximum load of 643 MPa and minimum load of 243 MPa. (**a**) Comparison of curves during the second loading cycle. Plastic deformation strain amplitude values are approximately 0.09% and 0.19% for CuCrZr and Cu+alumina, respectively. (**b**) Comparison of curves during the tenth loading cycle. Plastic deformation strain amplitude values are approximately 0.02% and 0.12% for CuCrZr and Cu+alumina, respectively.

In our second sequence of tests, we applied tensile and compressive loads of 643 MPa and 243 MPa, respectively, thus maintaining the maximum stress value from the first set of tests but increasing the compressive load, raising the stress amplitude by 20%. Increasing stress amplitude increased strain amplitude, but only marginally. Although no significant difference was observed between CuCrZr and Cu–alumina after the first loading cycle, the plastic deformation strain of CuCrZr after the second cycle was about half that of Cu–alumina (Figure 6). After the tenth loading cycle, the plastic deformation strain of CuCrZr was just one-fifth that of Cu–alumina. Our results demonstrated that, under the same tensile stress level and stress amplitude used for cyclic tests, CuCrZr exhibited considerably less plastic deformation strain than Cu–alumina.

### 3.3. Microstructure

In our two materials, CuCrZr and Cu–alumina, we observed both micro-sized and nanosized particles. Depending on their size and type, ionic bonded particles and metallic bonded particles behaved differently during deformation. Most particles in CuCrZr were deformable; most in Cu–alumina were non-deformable. Micro-sized particles were generally larger in CuCrZr than in Cu–alumina.

#### 3.3.1. Cu Strengthened by Deformable Particles

Light-microscopy images of solution-treated samples revealed a high density of pre-existing micron-scale and submicron-scale particles. These particles were formed during solidification and did not completely dissolve during solution treatment. The particles were uniformly distributed throughout the Cu matrix, both within grain interiors and at grain boundaries.

**Figure 7:**
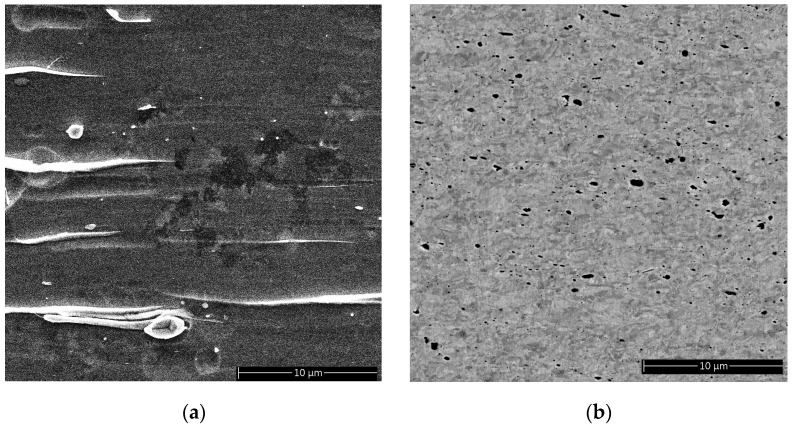
Comparison of SEM images of Cu strengthened by two types of particles. The samples are in wire form with the wire axis parallel to the micron bar in the images: (**a**) A CuCrZr sample prepared by electron-polishing. Regions with bright contrast are from particles enriched in both Cr and Zr. (**b**) Cu–alumina prepared by FIB. Regions with dark contrast are alumina.

Using SEM, we identified the presence of micro-sized particles in the deformed samples. These particles appeared in various forms: round, oval, rod, or needle. The size distribution was wide, ranging from 0.1 μm to several micrometers. Most of these particles contained Zr and a significant amount of Cr. EDS/SEM analysis showed that the atomic ratios of Cr/Cu for micro-sized particles (i.e., greater than 1 µm) ranged from 2:8 to 8:2, with higher Cr content in the larger particles. A few particles were also rich in Zr. We deduced that most of these particles had metallic bonding. Although the particles had a variety of shapes, most were elongated along the drawing direction (i.e., the wire axis). Therefore, these particles must have deformed along with the Cu matrix, creating various shapes (Figure 7).

EDS/TEM showed that the average atomic ratio of Cr/Cu in submicron-sized particles was about 2:8. Our TEM-EDS and SEM-EDS data demonstrated that the larger the particle size, the higher the Cr concentration. The Cu content in these particles was much higher than the expected solubility limit showed by the phase diagram of Cu in Cr.

**Figure 8:**
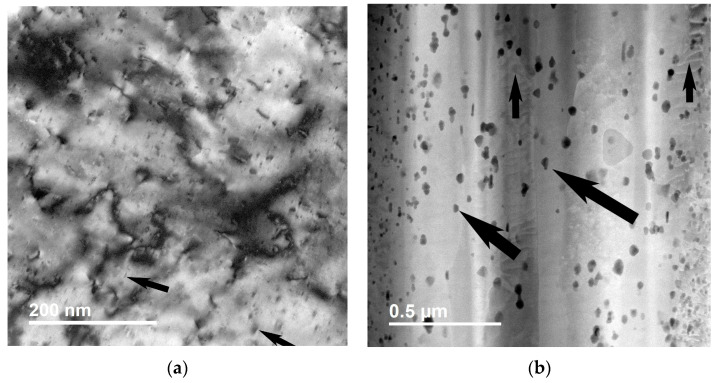
Comparison of microscopy images of Cu strengthened by two types of particles: (**a**) TEM image of a CuCrZr sample prepared by electron-polishing. Some strengthening particles are marked by arrows. Most nanosized particles are rich in Cr with a face-centered cubic structure. (**b**) STEM HAADF image showing Cu–alumina prepared by FIB. Some alumina particles are marked by larger arrows. Most nanosized alumina particles are in the γ phase. Some dislocations are marked by small arrows. The sample is in wire form with the drawing axis perpendicular to the micron bar in the images.

Using EDS/TEM, we observed nanosized particles that were rich in Cr (Figure 8). The size of the particles appeared to affect their crystallographic structure. Normally, a body-centered cubic crystallographic structure would be expected if the particles were sufficiently large, but in this case, most of these particles had the same crystallographic structure as the matrix—that of face-centered cubic.

**Figure 9:**
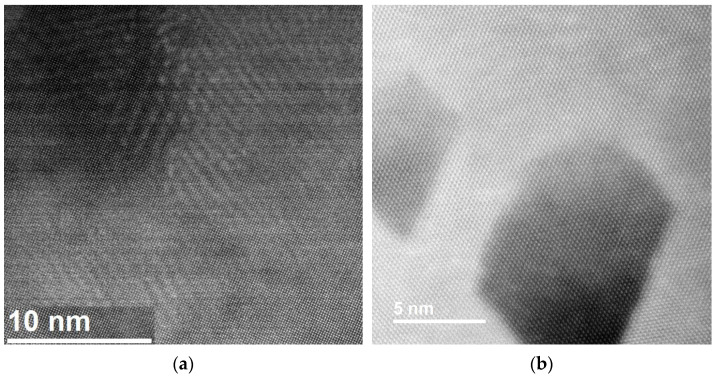
Atomic-resolution STEM HAADF image showing strengthening particles in Cu matrix. (**a**) A particle at the left top of the image in CuCrZr. (**b**) Two alumina particles in Cu–alumina. Both particles have a similar rhombic shape. The particle on the left has a near-atomic sharp edge that leads to stress concentration when the sample is under tension. The image was taken at <110> of the Cu matrix. The atomic columns of alumina are in darker contrast, while the atomic columns of the Cu matrix are in lighter contrast.

We examined strengthening particles at the atomic scale to understand the interfaces between the particles and the matrix. We determined that most interfaces were coherent. We observed no shape corners at the interface between the particles and the matrix (see Figure 9).

#### 3.3.2. Cu Strengthened by Non-Deformable Particles

In our Cu–alumina conductors, the non-deformable particles were alumina, which varied in size by a factor of 500 (from 10 nm to 5 µm.) As we saw in Cu-Cr-Zr alloys, different sizes of particles had differences in crystallographic structure and chemistry. The two most important particles were α and γ—the size of most of the α alumina particles was thousands of nanometers (micron scale), while γ alumina particles were in the nanometer scale (Cf. Figure 7 and Figure 8). We observed no plasticity in any particles.

We occasionally found micro-voids near larger micro-sized particles. We speculate that these larger particles had been added during the powder-oxidation fabrication process but had never fully reacted and decomposed to form nanosized particles.

We examined γ alumina particles at the atomic scale to understand the interfaces between these particles and the matrix. We determined that the interfaces were in an orientation of {111}_Cu_. Two of these interfaces met at an angle of 60 degrees, forming an arrow-like shape in one of the corners of the particle (see Figure 9).

## 4. Discussion

The range of conductivity in our samples at room temperature was only about 2% IACS, but at 77 K, the range was significantly greater. We observed up to 100% IACS higher electrical conductivity in Cu–alumina than in CuCrZr. We assumed that this conductivity difference between our two materials at 77 K could be attributed to one of three possibilities: (a) a difference in dislocation density, (b) a difference in the amount of dissolved alloying elements, or (c) a combination of the two. We settled on the second option because we were relatively sure that the dislocation densities of the two materials were identical, given that both materials had been deformed to similar deformation strain and had similar mechanical strength. Consequently, we attributed the lower conductivity of CuCrZr at 77 K to the expectation that a greater concentration of alloying elements would have been dissolved in the Cu matrix of CuCrZr than in the Cu matrix of Cu–alumina. If the presence of a higher percentage of dissolved alloying elements leads to greater solid solution strengthening, then solid solution strengthening can be expected to be consistently lower in Cu–alumina than in CuCrZr.

We found that at room temperature, in most of our samples, the higher the strength of any given conductor, the lower its conductivity. We did not find such a clear trend, however, in samples tested at 77 K.

Both micro-sized and nanosized particles were present in our conductors. Because the distance between the micro-sized particles was more than one magnitude higher than that between the nanosized particles, we deduced that nanosized particles must play a major role in strengthening the composites. Our data showed that, although particles with ionic interatomic bonding had greater hardness than those with metallic bonding, the strengthening effects of these two types of particles were almost identical.

Our close examination of stress–strain curves revealed that, in Cu–alumina, a higher level of internal stress developed within the material at the initial stage of deformation. We divided internal stress into two portions: macroscale and microscale. Our Cu–alumina composite conductors were made from (1) pure Cu cladding and (2) a Cu–alumina core. Because of the difference in flow stresses between these two components, incompatibility occurred during deformation, producing macroscale internal stress. We assumed that this additional stress was added to the microscale stress concentration that had already accumulated at the sharp corners of the γ-alumina particles. In CuCrZr, internal stress was lower, probably because (1) no cladding was required for making those conductors and (2) the strengthening Cr particles had almost the same crystallographic structure as the matrix (Figure 9).

We believe that the alumina particles in our conductors are strong enough to resist the motions of dislocations, so these dislocations can be expected to curve around the particles, as predicted by the theory of Orowan-strengthening. In that case, the shear stress for dislocations that curve around particles with volume fraction f and radius r can be described as follows:

S_o_ = CGbf^0.5^/r ln(2r/r_0_) (1)

where C is 0.093 for edge dislocation and 0.14 for screw dislocations, G is the shear modulus of Cu, b is the Burgers vector, and r_0_ is the radius of the dislocation core (where Hooke’s law is not applicable) [46].

In CuCrZr, most of the Cr particles are small enough and soft enough to be cut through by dislocations. The required shear stress can be described as follows:

S_s_ = 1.1є^3/2^ (rf/α)^0.5^/(Gb^2^) (2)

where є is the interface energy and *α* is 0.16 for edge dislocation and 0.24 for screw dislocation [47].

A comparison of Equations (1) and (2) indicates that when the size of the strengthening particle is small, alumina has a greater strengthening effect than Cr. The ratio of the two types of stress is:

S_s_/S_o_ = [r ln(2r/r_0_) є^3/2^ (r/α)^0.5^]/[ C/1.1 G^2^b^3^] (3)

Volume fraction f is not present in Equation (3), indicating that the relative strengthening effects of the two types of particles that occur in composite conductors have no relationship with f. Considering these two types of particles, it appears that those with ionic interatomic bonding provide a higher strengthening effect than those with metallic bonding. This was not necessarily true, however, for the longer-length conductors that we had made previously (Figure 3). We attributed the high probability of failure in those conductors to the buildup of stress concentration that had previously occurred near large ionic particles during the fabrication of those conductors.

The two types of conductors chosen for cyclic tests had almost identical mechanical strength at 77 K. Our cyclic test data reflected the impact of types of particles on samples under cyclic loading at 77 K. Samples strengthened by ionic particles appeared to need larger plastic deformation strain so that they could accommodate the same magnitude of load as could be accommodated by samples strengthened by metallic particles, even though the latter had slightly lower UTS.

In most cyclic tests, researchers perform symmetrical fatigue tests; i.e., the tensile load is the same as the compressive load. Most conductors in our case, however, are subjected to higher levels of tensile load than compressive load; i.e., we conducted our cyclic tests with higher tensile stress than compressive stress.

We and other researchers have studied, not only Cu strengthened by particles with either metallic bonding or ionic bonding, but also Cu strengthened by particles with atoms bonded by van der Waals’s force (e.g., carbon nanotubes or graphene). Some researchers have speculated that carbon nanotubes or graphene might be capable of producing a higher strengthening effect than the metallic bonding or ionic bonding particles previously studied. Published results, however, have so far indicated that conductors strengthened by metallic bonding or ionic bonding particles remain much stronger than those strengthened by carbon nanotubes or graphene [48,49].

## 5. Conclusions

Under cyclic loading with the same maximum and minimum loads, conductors strengthened by metallic particles exhibited less plastic deformation strain than those strengthened by ionic interatomic bonding particles. During the initial loading cycle, the plastic deformation strain of conductors strengthened by metallic particles was about 50% lower than the strain of conductors strengthened by ionic interatomic bonding particles. In subsequent loading cycles, the plastic deformation strain of conductors strengthened by metallic particles was cumulatively even lower, despite the fact that ionic particles are much harder than metallic ones. Although increasing stress amplitude without increasing maximum stress did increase the strain amplitude, the overall trend remained unchanged. We attribute this anomaly to the magnitude of stress concentration near the particles, particularly the micron-sized ones. Conductors strengthened by ionic particles have limited plasticity in comparison with conductors strengthened by metallic particles, which are much more malleable. Atomic-scale shape corners induce higher stress concentration in conductors strengthened by ionic particles. This limitation is not necessarily intrinsic. Developing composites without large ionic particles and shape corners will significantly improve the ductility of these conductors.

## Figures and Tables

**Table 1:** Comparison of mechanical properties of two composites.

Sample No.	Temp. (K)	Modulus (GPa)	Strength (MPa)		Reduction	Conductivity
			Yield	Tensile	of Area	% IACS
Al60-2307-94	295	113	519	557	47	83.8
CuCrZr_21017	295	117	563	586	54	84.2
Al60-2307-94	77	134	627	716	12	373.8
CuCrZr_21017	77	134	655	708	53	309.5

## Data Availability

The original contributions presented in this study are included in the article. Further inquiries can be directed to the corresponding author.
